# No association between the aluminium content of trabecular bone and bone density, mass or size of the proximal femur in elderly men and women

**DOI:** 10.1186/1471-2474-7-69

**Published:** 2006-08-23

**Authors:** Hans-Olov Hellström, Bengt Mjöberg, Hans Mallmin, Karl Michaëlsson

**Affiliations:** 1 Department of Orthopaedics, Uppsala University Hospital, Sweden; 2 Västra Vallgatan 29, SE-271 35 Ystad, Sweden

## Abstract

**Background:**

Aluminium is considered a bone toxic metal since poisoning can lead to aluminium-induced bone disease in patients with chronic renal failure. Healthy subjects with normal renal function retain 4% of the aluminium consumed. They might thus also accumulate aluminium and eventually be at risk of long-term low-grade aluminium intoxication that can affect bone health.

**Methods:**

We therefore examined 62 patients with femoral neck fractures or osteoarthritis of the hip (age range 38–93), with the aim of examining whether aluminium in bone is associated with bone-mineral density (BMD), content (BMC) or width of the femoral neck measured by dual-energy X-ray absorptiometry (DXA). During operations bone biopsies were taken from the trabecular bone of the proximal femur. The samples were measured for their content of aluminium using a mass spectrometer.

**Results:**

No significant association between the aluminium content in bone and femoral neck BMD, BMC or width could be found after multivariate adjustment.

**Conclusion:**

Our results indicate that the accumulated aluminium content in bone during life does not substantially influence the extent of osteoporosis.

## Background

Osteoporosis is a common disease in the elderly, and is characterised by a generalised reduction in bone-mineral density (BMD) or mass (BMC), microarchitectural deterioration of bone tissue and an increased risk of low-energy fractures. The disease is divided in two groups: primary and secondary osteoporosis. Primary osteoporosis is becoming more prevalent with ageing and is influenced by many different environmental factors and lifestyles, as well as genetic disposition [1-9]. Aluminium, one potential environmental factor of interest for bone disease, is the third most common element in the earth's crust and the most abundant metal (8%), and is widely utilised in industry. Owing to its low solubility and because of its deposition as sediment in the form of aluminium hydroxide, only small amounts of aluminium in solution are usually found in water (< 10 μg/l). However, aluminium levels are higher in an acidified environment due to acid rain or in water treated with aluminium sulphate, i.e. in waterworks for the chemical removal of particles in drinking water. Aluminium is also naturally present in many foods, and total dietary intake has been estimated at 4–9 mg Al/day [10-14]. While aluminium normally enters the body through the diet, intoxication by inhalation of aluminium-containing gas is a rare, but occasionally occurs in the aluminium-processing industries. On average, 4% of the aluminium content of the diet is retained by intestinal absorption, and might partially be accumulated in bone, the main storage site of aluminium [15,16]. In the elderly and in patients with Alzheimer-type pathology, the intestinal absorption of aluminium is increased [17,18], thus augmenting the amount of aluminium stored in bone. In the bloodstream, most aluminium is eliminated through the kidneys.

Given its wide use and occurrence, the conceivable harmful potential of aluminium for negative biological consequences in humans is high. It is well known in patients with chronic renal failure that aluminium poisoning may lead to three types of disorder: aluminium-induced bone disease (AIBD), microcytic anaemia and encephalopathy [19,17,18,28], but since healthy subjects with normal renal function also retain aluminium, they potentially risk long-term accumulation and low-grade aluminium intoxication [15,29-31]. Furthermore, osteomalacia-like bone disease has been reported in patients who required long-term total parenteral nutrition (TPN) [32-34] owing to the aluminium contamination of the protein source used.

The aim of this study was to examine whether the aluminium concentration in bone is associated with BMD, BMC or size of the proximal femur in middle-aged and elderly men and women.

## Methods

In total, 62 patients – 41 women and 21 men – with a mean age of 72, range 38–93, were included in this study, and all characteristics are shown in Table 1. They were treated at either of the two hospitals in the county of Uppsala. They were admitted to hospital for arthroplasty because of osteoarthritis of the hip (ICD X code M161, n = 34, mean age 63 years; range 38–90 years) or for hip fracture (ICD X codes S720 or S721, n = 28, mean age 82 years; range 72–93 years). Of these hip-fractures cases, 13 had a diagnosis of dementia on admission to hospital, while 15 patients had no reported mental illness. None of the patients operated on for osteoarthritis were recognised as being demented.

Creatinine in serum was measured kinetically as a creatinine-picrate complex based on a modified Jaffe's reaction in a spectrophotometer. The coefficient of variation (CV) was 2.1% and the normal reference interval was set to 60–106 μmol/l. Creatinine clearance in ml/minute was estimated using the Cockroft and Gault equation [35], including the variables serum creatinine, body weight, height, age and sex.

During the operations in all cases, bone biopsies from the trabecular bone of the proximal femur (i.e. Trochanter major, after drilling the hole for the osteosynthesis screws or after preparation for the prosthetic femur stem in cases of arthroplasty) were taken using an aluminium-free instrument. The mean weights of the samples were 300 mg (range 29–600 mg). The bone samples were immediately put in sealed polyethylene test tubes, frozen and stored at -20°C until analysis. Prior to analysis, after drying at 120°C for 48 hours, the bone samples were weighed. Thereafter, the biopsies were decomposed using ultra-pure nitric acid in a quartz tube, and an internal standard (Indium) was added, diluted with high-purity water (with a resistivity of more than 18 MΩ-cm). The samples were then introduced into an inductively coupled mass spectrometer (Perkin-Elmer Elan 6000) and measured for their content of aluminium. All handling of the samples was in a clean room. Quality control was performed using a reference material (IAEA H-8 Animal bone) in every fifth sample randomly distributed in the measurement series. The coefficient of variation (CV) for the method was 4.7% [36].

Femoral-neck and total hip BMD (g/cm^2^), BMC (g/cm) together with neck width (cm) and area (cm^2^) measurements were performed using dual energy X-ray absorptiometry (DPX-L™, Lunar Co, Madison, Wi, USA) of the contralateral unfractured/nonoperated proximal femur. The scans were performed within one week of the fracture event, or in arthroplasty cases 1–2 days before the operation. The equipment's precision error expressed as CV was evaluated using a spine phantom and was less than 1%.

The Ethics Committee of the Medical Faculty of Uppsala University approved the study.

### Statistical analysis

The measured aluminium levels of the bones displayed a skewed distribution. In all analysis we therefore used aluminium values transformed to natural logarithms – values which in turn were normally distributed (Shapiro-Wilk test p = 0.45). The crude and the multivariate adjusted results with sex, age (by 10-year groups), body-mass index (kg/m^2^, continuous), height (continuous), demented hip-fracture cases (dichotomous) and non-demented hip-fracture cases (dichotomous) by linear regression modelling are presented. To detect non-linear associations we used the mean multivariate adjusted BMD and BMC values by tertiles of aluminium content of the bones, using the GLM (general linear models) procedure in the SAS package (version 8, SAS Institute, Cary, NC, USA).

## Results

All samples contained aluminium, ranging from 58 to 6435 ng/g dw. We found an increase in aluminium content of bone with age (R^2 ^= 0.3, p < 0.0001). The average aluminium values, adjusted for age, were similar in men and women (p = 0.47).

**Figure 1:**
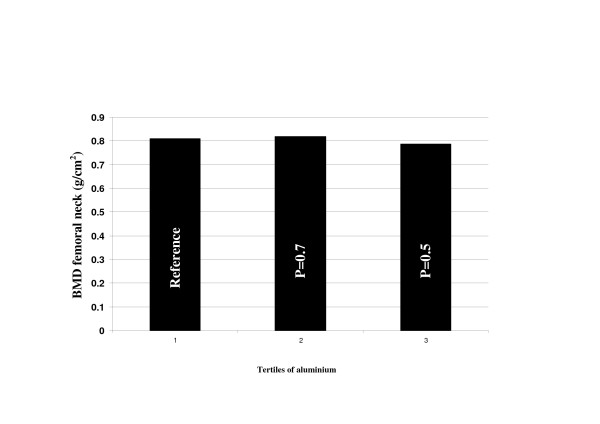
**Tertiles Al-BMD**. Average adjusted bone-mineral density and content values of the femoral neck by tertiles of aluminium content of bone. Included in the multivariate model were sex, age (by 10-year groups), body-mass index (continuous), height (continuous), creatinine clearance (continuous), demented hip fracture cases (dichotomous) and non-demented hip-fracture cases (dichotomous).

**Figure 2:**
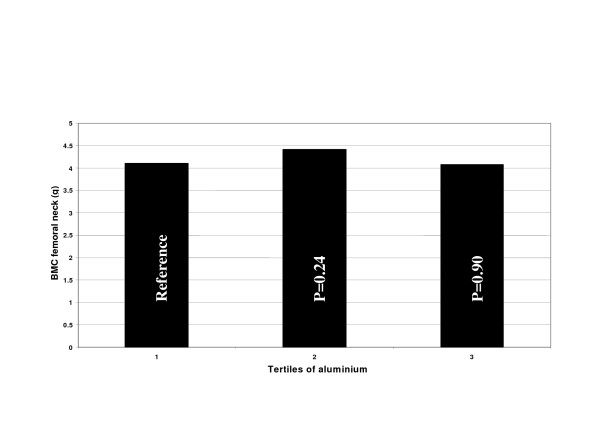
**Tertiles Al-BMC**. Average adjusted bone-mineral density and content values of the femoral neck by tertiles of aluminium content of bone. Included in the multivariate model were sex, age (by 10-year groups), body-mass index (continuous), height (continuous), creatinine clearance (continuous), demented hip fracture cases (dichotomous) and non-demented hip-fracture cases (dichotomous).

Using a crude linear-regression analysis model, we found a strong association between aluminium and BMD as well as with BMC, but after multivariate adjustment including age these associations did not remain significant (Table 2). Furthermore, there were no differences in adjusted mean BMD or BMC values between tertiles of aluminium content in bone (Figures 1 and 2). In addition, we could neither find an association between aluminium in bone and bone density nor any association regarding mass within subgroups such as demented or non-demented participants among those with hip fracture or osteoarthritis patients or in any gender. No associations were found between the aluminium content in bone and femoral-neck width or area (Table 2).

## Discussion

Our cross-sectional study suggests no major association between the aluminium content of bone and BMD, BMC or bone size, overall or in subgroups such as demented or fractured cases. We are not aware of any previous studies investigating whether aluminium might exert negative effects on bone density, mass or size in humans. There are, however, scientists who have examined the effect of aluminium on bone cells in vitro, but the results are somewhat conflicting, indicating that aluminium could have negative or positive effects on bone metabolism, or none at all. In fact, in osteopenic rats with normal renal function aluminium is able to induce bone formation [37], and comparable results have been observed in dogs [38-40]. Actually, in the former study both high osteoblastic activity and high osteoclastic activity were observed simultaneously, representing a high bone-turnover rate, which is an independent risk factor of fractures in humans [41]. There are also, on the contrary, animal studies in which aluminium has been proven to inhibit osteoblastic activity and have a negative effect on bone mineralisation [42,43] Indirectly, aluminium also seems to have a negative effect on bone by interfering with parathyroid hormone (PTH) release and maybe the synthesis of PTH, possibly by elevating the serum calcium level, and it also seems to alter the homeostasis between calcium and phosphorus [23,44-46]. These paradoxical effects in experimental and animal studies of the metal, i.e. both trophic osseous actions and bone-toxic actions, are enigmatic and await further explanation.

Despite the experimental indications, no human studies have shown that aluminium could induce bone formation. On the contrary, use of aluminium cooking pots increases the risk of hip fractures [47] Two small pilot studies [31,48] have analysed the aluminium content in bone in patients with a hip fracture. The former study displayed that hip fracture cases with Alzheimer's disease had higher aluminium levels in bone than non-demented hip fracture cases, a finding that was not confirmed in the latter survey. Our recent larger case-control study displayed no significant increased risk of hip fracture with high bone levels of aluminium as well as it was a exponential increase in aluminium content in bone with age [49] Since age is also an important covariant in our present analysis with BMD and BMC as outcomes we have chosen to also mention the positive association with aluminium content in bone with age.

Some discrepancies also exist between previously reported normal aluminium contents in bone [50-52] that are probably due to differences in study design and chemical processing such as contamination of aluminium or other interfering metal ions, and probably the choice of analytical method used. Most variability of the reported normal content of aluminium in bone is probably due to contamination, which is a serious problem when dealing with samples containing low concentrations of aluminium because of the ubiquitous nature of the element. Thus the lowest recorded values are probably the most correct ones [53] This observation is supported by the results of one of our previous studies using a meticulous analytical technique on a large group of humans. We observed that the values for younger individuals and individuals in midlife were close to the lowest values previously published in the literature [49].

Selection bias is a potential limitation of our study. It can be argued that the patients in the study group are not truly representative of the population since they were admitted to the hospital and included in the study because of injury or osteoarthritis, and thus cannot be considered entirely healthy. Chronic low-grade aluminium intoxication is, however, not expected to play a role in the development of osteoarthritis, and we also consider it unlikely that our cases were accidentally selected as a result of the aluminium status of the bone.

Our crude results indicated a strong negative association between the aluminium content of bone and the amount of bone in the proximal femur. These associations disappeared after multivariate adjustment, especially age adjustment. The confidence intervals for the adjusted results indicate that at least half of the crude association was explained by confounding. Our sample size was sufficient for detection of moderate effects. According to our multivariate adjusted confidence intervals of the parameter estimates, we had a 95% chance of detecting a 2–3% difference in bone mineral density or mass of the proximal femur for each standard deviation change in bone aluminium.

None of the patients were excluded from the analysis, nor were any of our participants treated for renal failure. Even though renal failure and dialysis are associated with higher bone aluminium levels [54,55], a more subtle deterioration in kidney function is not known to induce aluminium accrual in bone. Since none of our participants suffered severe renal failure or had been treated with haemodialysis we consider it unlikely that renal function explains the higher aluminium values among the oldest individuals in our study. The accuracy of the diagnosis of dementia is a possible shortcoming, since it is based on medical records. A geriatric specialist made the diagnosis, therefore it can be assumed that the diagnosis is fairly reliable.

## Conclusion

In this study we have not been able to show any significant association between the aluminium content in bone and the degree of BMD, BMC or bone size of the proximal femur measured using DXA. Thus, our study does not support the hypothesis that aluminium is involved in the pathogenesis of osteoporosis.

## Competing interests

The author(s) declare that they have no competing interests.

## Authors' contributions

All authors participated in the design of the study and drafting the manuscript.

All authors has read and approved the final manuscript.

## Pre-publication history

The pre-publication history for this paper can be accessed here:



## Figures and Tables

**Table 1:** Characteristics of the participants (n = 62).

	**Mean (SD)**	**Range**
**Age (years)**		38 – 93
**Aluminium ng/g dw**	1262 (1377)	58 – 6435
**log Aluminium**	2.9 (0.4)	1.8 – 3.8
**Weight (kg)**	70 (14)	44 – 102
**Height (m)**	1.67 (0.09)	1.49 – 1.9
**BMI (Kg/m^**2**^)**	24.6 (3.4)	19 – 32
**Creatinine (μmol/l)**	92 (20)	64 – 187
**Creatinine clearance (ml/minute)**	50 (19)	19 – 93
**BMD femoral neck (g/cm^**2**^)**	0.81 (0.18)	0.46 – 1.25
**BMC femoral neck (g)**	4.20 (1.23)	1.96 – 7.25
**BMD total hip (g/cm^**2**^)**	0.86 (0.19)	0.54 – 1.29
**BMC total hip (g)**	30.3 (8.1)	15.0 – 49.7
**Width femoral neck (cm)**	5.2 (0.7)	3.0 – 6.6
**Area femoral neck (cm^**2**^)**	34.9 (4.0)	26.6 – 46.4

**Table 2:** Results of linear-regression analysis using logarithmically transformed aluminium values as independent variables, and bone-mineral density (BMD) and content (BMC) of the total hip and femoral neck as dependent variables.

		**Univariate model β (SE)**	**95% confidence interval of β**	**p-value**	**Multivariate model β (SE)**	**95% confidence interval of β**	**p-value**
	Unit of exposure						
**BMD femoral neck (g/cm^**2**^)**	**	-0.098 (0.02)	-0.137; -0.059	<0.0001	-0.023 (0.015)	-0.054; 0.008	0.14
	***	-0.039 (0.008)	-0.055; -0.024	<0.0001	-0.010 (0.006)	-0.022: 0.003	0.13
**BMD total hip (g/cm^**2**^)**	**	-0.088 (0.021)	-0.131; -0.045	0.0001	-0.013 (0.015)	-0.044; 0.018	0.39
	***	-0.035 (0.009)	-0.052; -0.018	0.0001	-0.006 (0.007)	-0.020; 0.007	0.36
**BMC femoral neck (g/cm)**	**	-0.56 (0.14)	-0.84;-0.29	0.0001	-0.09 (0.11)	-0.32; 0.13	0.41
	***	-0.22 (0.05)	-0.33;-0.12	0.0001	-0.04 (0.05)	-0.14; 0.05	0.38
**BMC total hip (g/cm)**	**	-3.00 (0.95)	-4.91; -1.10	0.003	-0.31 (0.63)	-1.56; 0.95	0.63
	***	-1.20 (0.38)	-1.96; -0.44	0.003	-0.17 (0.28)	-0.74; 0.41	0.57
**Width femoral neck (cm)**	**	-0.076 (0.083)	-0.242; -0.089	0.36	0.025 (0.084)	-0.14; 0.19	0.77
	***	-0.031 (0.033)	-0.097; 0.036	0.36	0.010 (0.034)	-0.058; 0.078	0.77
**Area femoral neck (cm^**2**^)**	**	0.07 (0.51)	-0.95; 1.09	0.89	0.26 (0.34)	-0.42; 0.95	0.44
	***	0.03 (0.20)	-0.38; 0.44	0.89	0.11 (0.14)	-0.17; 0.38	0.44

* Adjusted for sex, age (by 10-year groups), body-mass index (continuous), height (continuous), creatinine clearance (continuous), demented hip-fracture cases (dichotomous) and non-demented hip-fracture cases (dichotomous) adjusted the analysis.

β;parameter estimate, SE; standard error

** per unit of log-transformed aluminium

*** per SD of log-transformed aluminium
